# Superficial Characteristics of Titanium after Treatment of Chorreated Surface, Passive Acid, and Decontamination with Argon Plasma

**DOI:** 10.3390/jfb9040071

**Published:** 2018-12-11

**Authors:** María Rizo-Gorrita, Irene Luna-Oliva, María-Angeles Serrera-Figallo, Daniel Torres-Lagares

**Affiliations:** Dental School, University of Sevilla, 41009 Seville, Spain; marrizgor@alum.us.es (M.R.-G.); irene_ire337@hotmail.com (I.L.-O.); maserrera@us.es (M.-A.S.-F.)

**Keywords:** Titanium, surface roughness, dental implant, surface composition, microtopography, scanning electronic microscope, cell culture, RT-PCR

## Abstract

(1) Background. Titanium is characterized by its biocompatibility, resistance to maximum stress, and fatigue and non-toxicity. The composition, surface structure, and roughness of titanium have a key and direct influence on the osseointegration processes when it is used in the form of dental implants. The objective of the present study is to characterize, at chemical, superficial, and biological levels, the result of the application of the sandblasted with large-grit and acid-etched (SLA) treatment consisting of coarse-grained and double-passivated acid blasting with subsequent decontamination with argon plasma on the surface of titanium implants type IV. (2) Methods. Four Oxtein^®^ dental implants (Zaragoza, Spain) were investigated with the following coding: Code L63713T (titanium grade IV, 3.75 mm in diameter, and 13 mm in length). The surface of the implants was SLA type obtained from coarse-grained, double passivated acid, and decontaminated with argon plasma. The samples were in their sealed packages and were opened in our laboratory. The X-ray photoelectron spectroscopy (XPS) technique was used to characterize the chemical composition of the surface, and the scanning electronic microscope (SEM) technique was used to perform topographic surface evaluation. Cell cultures were also performed on both surfaces. (3) Results. The superficial chemical analysis of the studied samples presented the following components, approximately, expressed in atomic percentage: O: 39%; Ti: 18%; C: 39%; N: 2%; and Si: 1%. In the same way, the topographic analysis values were obtained in the evaluated roughness parameters: R_a_: 1.5 μm ± 0.02%; R_q_: 1.31 μm ± 0.33; R_z_: 8.98 μm ± 0.73; R_p_: 5.12 μm ± 0.48; R_v_: 3.76 μm ± 0.51; and R_c_: 4.92 μm ± 0.24. At a biological level, the expression of osteocalcin was higher (*p* < 0.05) on the micro-rough surface compared to that machined at 48 and 96 h of culture. (4) Conclusions. The data obtained in our study indicate that the total carbon content, the relative concentration of titanium, and the roughness of the treatment performed on the implants are in agreement with those found in the literature. Further, the roughness of the treatment performed on the implants throws a spongy, three-dimensional surface suitable for bone growth on it. The biological results found are compatible with the clinical use of the surface tested.

## 1. Introduction

After 1969, with the discoveries of Brånemark, biomedicine, and of course dentistry, entered one of the greatest revolutions in its history. The concept of osseointegration appeared, which was based on the capacity of titanium as an artificial device capable of interacting with the organism and allowing adequate long-term interaction with the bone [1].

Titanium is characterized by its biocompatibility, resistance to maximum tension and fatigue, non-toxicity. However, in the human mouth, titanium can be in contact with various ions, such as fluoride from toothpastes, with H_2_O_2_ (produced by bacteria or leukocytes), or with acids such as lactic acid, which have been cited as the causes of titanium corrosion [2,3]. To determine the physical-chemical characteristics of titanium and its influence on the response of tissues, two types of study were conducted. One focused on the chemical properties of surface, and the other aimed at analyzing the topography of it. Both characteristics affect cellular and tissue behavior. Other materials have also been used for the purpose of inserting dental implants [4,5].

Regarding the chemical properties, within the commercially pure titanium we find four grades that depend on the content of impurities (oxygen, nitrogen, carbon, hydrogen, and iron) that control and can vary the mechanical properties of it. Other elements were also observed, including phosphorus, calcium, silicon, and chlorine. They were usually presented in low percentages and may be derived from washing, or as the case may be, from some of the processing phases.

Within the four grades encompassed by commercially pure titanium (CpTi), each contains amounts of titanium greater than 98%. These grades are also called unalloyed grades. The grades of alloy titanium encompass grades 5 and above. The titanium grade 5 (Ti_6_Al_4_V) consists of an alloy of titanium (90%), aluminum (6%), and vanadium (4%). The pure titanium grades rise as oxygen concentration increases, presenting, as a general rule, values between 0.18% and 0.40% (grade 1, 0.18%; grade 2, 0.25%; grade 3, 0.35%; and grade 4, 0.40%). In addition to oxygen, CpTi titanium also contains small percentages of nitrogen, hydrogen, iron, and carbon. By increasing the concentration of oxygen, substantial improvements in breaking stress are obtained. On the other hand, chemical composition depends on the type of Ti alloy production technique [6].

In recent years many different surface treatments have appeared to increase the roughness of dental implants, as it is known that the appearance of roughness on the surface of the implant favors and increases osseointegration [7,8]. The topographic modification of the surface structure of the implant allows greater adhesion and proliferation of the osteogenic cells, thus creating mineral deposits that will form new bone over time, favoring greater and faster osseointegration.

Roughness can be considered the most important surface property for increasing and improving tissue response to a dental implant, as has been demonstrated in both in vivo and in vitro studies [9].

Three primary processes have been described in which the surface structure of the implant is modified. First are those intended for surface cleaning and/or removal of the native surface layer, such as cleaning with solvents (aliphatic hydrocarbons, alcohols, ketones, or chlorinated hydrocarbons, for instance) and polishing. In the second and third place are the modification of topographic surface structures of the implant by methods of addition or subtraction of material. Sandblasting, acid passivation, and titanium plasma projection are included in this group [10,11].

The roughness of the surface is a key factor in osseointegration and in the long-term success of the implant. Several studies have shown the importance of surface modification at the micrometer level due to the significant improvement in bone formation at this level [12,13,14]. Abrahamsson demonstrated that double acid passivation resulted in greater bone-implant contact compared to machined surfaces, and this is why techniques have been developed that incorporate it systematically, such as the sandblasted with large-grit and acid-etched (SLA) surface [15,16,17,18].

Oxtein^®^ implants have been subjected to surface modification procedures based on coarse-grain blasting (250–500 μm) and double acid passivation. Such surfaces, as mentioned above, are usually referred to in the literature as SLA.

This term was introduced by Buser in 1991 and is based on a coarse-grain blasting technique with aluminum oxide particles that generate a macrorugosity on the surface of the implant [15]. Next, acid passivation is carried out to eliminate surface areas damaged by the blasting and to refine the characteristics of surface roughness [12]. In the case of Oxtein^®^ implants, it is carried out using a mixture of HCl with H_2_SO_4_ at a high temperature for several minutes. This procedure creates micro-wells superimposed on the surface of rough sandblasting, which results in characteristic micro-roughness.

After blasting, the reactivity of the surfaces against the etching solutions are different, and remarkable variations in roughness values can be observed. Thanks to the combination of both techniques, topographies are observed at different scales within the same surface [12]. In a comparative study of surfaces, Lukaszewska-Kuska et al. demonstrated that physical and chemical modifications of the surface of titanium (Ti) change its microstructure and increase its roughness [17].

In 2004, Albrektsson and Wennerberg classified implants into four categories according to the average roughness of their surface (S_a_): Smooth (S_a_ < 0.5 μm); minimally rough (S_a_ between 0.5–1.0 μm); moderately rough (S_a_ between 1.0–2.0 μm); and rough (S_a_ > 2.0 μm) [16]. Several authors have suggested that the optimum roughness value is in the range of 1–2 μm, as this provides an optimum degree of roughness to promote osseointegration, compared to the smoother or rougher surfaces [19]. The best clinical results have been obtained with moderately rough surfaces (S_a_ between 1.0–2.0 μm). In most studies using the techniques of blasting and acid passivation, a moderate roughness is obtained (S_a_ between 1.0–2.0 μm) [12,13].

The SLA surface presents its crystallographically oriented limits and a honeycomb structure with honey pores with a size of 1–3 μm, as observed by Kang et al. in their study [20].

In their comparative study of surfaces, Mendoza-Arnau et al. obtained a mean rugosity (R_a_, arithmetical mean deviation of the assessed profile) of 2–4 μm for SLA. This was characterized as moderate porosity with frequent and smooth peaks and valleys and with a homogeneous abrasion all based on the interpretation of the values R_v_ (maximum valley depth), R_p_ (maximum peak height) and R_t_ (maximum height of the profile) [21].

However, using the same procedure to characterize the surface, the roughness values obtained will not be the same according to the implant and titanium type used by the manufacturer, as demonstrated by Rosa et al. [22]. In their study, they observed that the results differed in average roughness and surface patterns. In blasting, characteristics of the surface depend on the type of particle used (for example Ti or Al_2_O_3_), particle size, hardness, and impact velocity. In acid etching, the factors to be taken into account are the type of acid, temperature, and immersion time [16]. This justifies the study and characterization of the multiple surfaces that can be found in the current implantology under the acronym SLA.

Other authors have evaluated the influence of the acid passivation time and the temperature of the solution with respect to the composition and surface characteristics of Ti grade IV discs. The surfaces with the highest roughness were those with the highest temperature and the longest immersion time, with temperature contributing most to the variation in the mean values of the surface roughness parameters when compared to the immersion time [23].

On the other hand, to improve cell adhesion and therefore the osseointegration of the implant, the surface must be clean and decontaminated. As commercial implant surfaces can be contaminated during manufacturing processes by being in contact with different tools or substances or during the acid passivation process, treatments such as the application of argon, helium, or oxygen plasma are carried out [23].

In the case of Oxtein^®^ implants, the cleaning of carbon impurities from the surface of the implant has been done through the application of argon plasma, which is a widely used and reproducible surface treatment. It is based on the application of a flow that creates a gaseous atmosphere that is not reactive at room temperature, which contains different elements, such as ions or electrons that are accelerated in an electric field and impact against the surface of the implant. This bombing allows the elimination of contaminants [24].

The objective of the present study is to characterize, at both chemical and superficial levels, the results of the application of the SLA treatment consisting of coarse-grained and double-passivated acid blasting with subsequent decontamination with argon plasma on the implant surface of titanium type IV from Oxtein^®^. Through this study, it will be possible to confirm that the composition, roughness, and biological properties detected in the topography of the surface is clean and in accordance with the values accepted and used for surfaces of dental implants for clinical use in humans.

## 2. Results

### 2.1. Chemical Analysis of the Surface

The superficial chemical analysis performed by XPS reflects that the two samples studied have the following components, expressed in atomic percentage:

- Sample 1: O: 39.4%; Ti: 18.1%; C: 39.4%; N: 1.8%; Yes: 1.0%; S: 0.2%.
- Sample 2: O: 39.2%; Ti: 18.0%; C: 39.9%; N: 1.9%; Yes: 0.9%; S: 0.1%.

**Figure 1:**
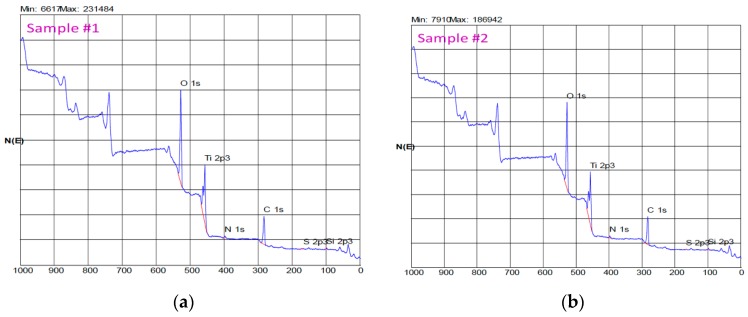
(**a**) EDS (Energy-dispersive X-ray spectroscopy) spectrum of one of the samples studied. (**b**) EDS spectrum of one of the samples studied.

The combined energy data obtained are shown in Figure 1a,b. The surface spectrum of both reveals higher peaks in Ti 2p, O 1s, and C 1s. The dominance of the titanium and oxygen signals show that the surface is mainly composed of a layer of titanium oxide based on TiO, TiO_2_, and TiO_3_. The signal obtained from carbon is mainly due to surface contamination by molecules containing carbon, which is to be expected on surfaces exposed to the atmosphere [25].

### 2.2. Surface Topography (SEM)

SEM analysis was performed to evaluate the surface topography of the Oxtein^®^ implants. The main findings are described below.

**Figure 2:**
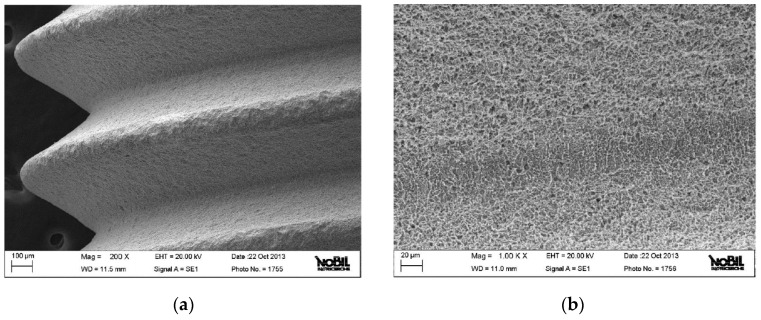
(**a**) SEM image at low resolution of the implant surface. (**b**) SEM image at medium resolution of the implant surface.

**Figure 3:**
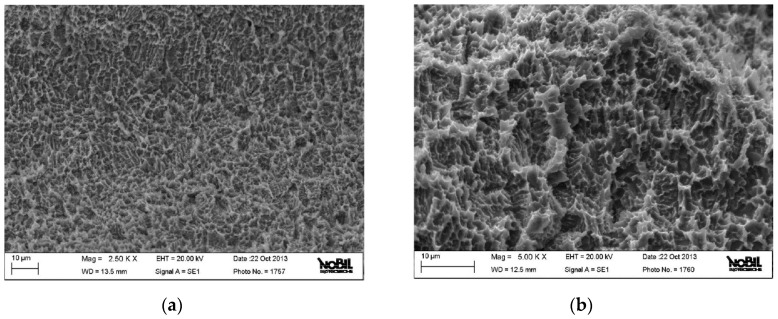
(**a**) SEM image at medium resolution of the implant surface. (**b**) High-resolution SEM image of the implant surface.

The images observed at low magnification show a homogenous surface with a good surface finish without the presence of residual particles of contamination according to the result with XPS (Figure 2a). Other larger extensions show the details of the topography of the surface at the micrometer level (Figure 2b and Figure 3a). The structure is porous with facets and wells caused by blasting and acid passivation. In addition, the surface presents crystallographically oriented limits, which are characteristic after passivation (Figure 3b).

In the topographic analysis performed, the values obtained from the roughness parameters evaluated on twelve points in both samples are as follows: R_a_: 1.49 μm ± 0.15%; R_q_: 1.27 μm ± 0.12; R_z_: 8.58 μm ± 0.68; R_p_: 5.10 μm ± 0.72; R_v_: 3.49 μm ± 0.34; and R_c_: 4.84 μm ± 0.52.

**Figure 4:**
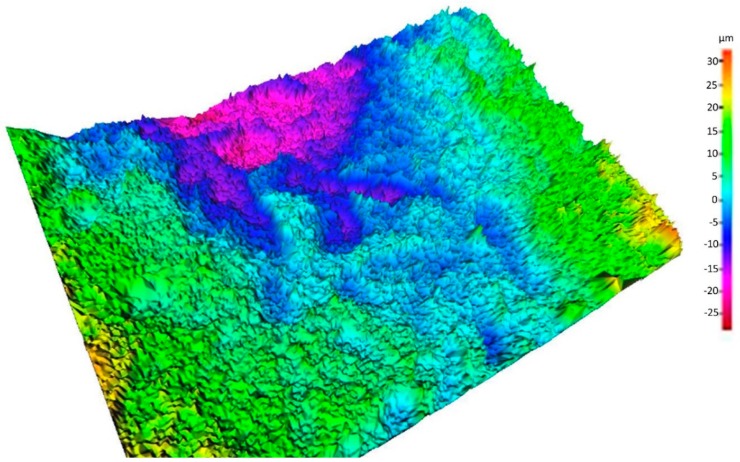
Map in three-dimensional color of the implant surface studied.

A pseudo-colored, three-dimensional reconstruction of the surface roughness of the implant obtained by SSEM is shown in Figure 4.

### 2.3. Cell Culture and RT-PCR Measurement

**Figure 5:**
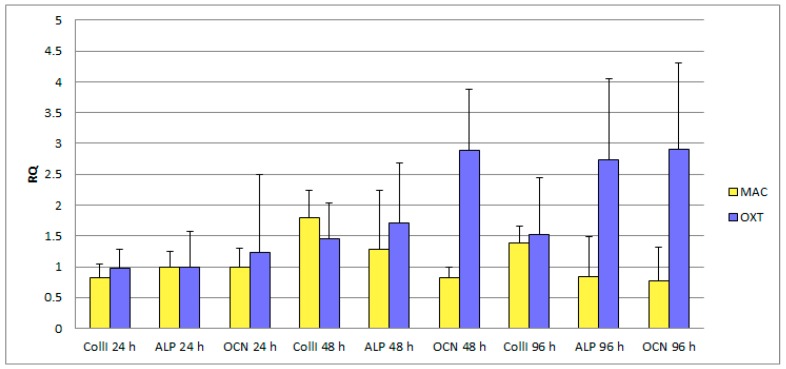
Results of RT-PCR measurements showing expression by SaOS 2 osteoblast-like cells of some osteogenic marker (Collagen I = CollI, Alkaline Phosphatase = ALP, and Osteocalcin = OCN) at different time points on microrough (OXT) and machined (MAC) surfaces. Differences between values MAC vs OXT are significantly different with *p* < 0.05 for OCN at 48 and 96 h, and ALP at 96 h. Remaining data are not significantly different.

Results of RT-PCR experiments are shown in Figure 5. In particular, the graph shows fold expression of genes codifying for CollI, ALP and OCN, comparing at three different time points data obtained from SaOS2 cells cultured on machined (MAC) or microrough (OXT) surfaces. While at the first time point (24 h), no significant differences are detected, expression of OCN at 48 h and of both ALP and OCN at 96 h are significantly higher on the microrough surface. This result is in agreement with existing literature on stimulation of osteoblast differentiation by microrough surfaces and confirms that, in the present case, the surface topography described in previous sections leads to overexpression of osteogenic markers by SaOS2 cells, as compared to machined surfaces.

### 2.4. MTT (Cell Proliferation Test) Measurement

Results of MTT (cell proliferation test) measurement are reported in the Table 1 (data are reported as absorbance values at 570 nm). Data show a higher initial adhesion on OXT surface (*p* < 0.05), while no differences are detected at longer time points. The measured absorbance values increase with time, following cell growth on the disks surface. Basically, data show that both surfaces support cell adhesion and growth. In this respect, Ti is a paradigmatic biocompatible material, and the MAC surface, which is pure Ti without any putative side-effect due to treatment, can be taken as a negative control in conventional cytotoxicity tests and in the assessment of cell viability and health. Thus, a further information that is gathered from the present data is that the OXT surface behaves as the negative control in terms of cell viability, that has no unexpected side effects on cell health, which are introduced by the treatment and cleaning procedures.

## 3. Discussion

In this study, the surface characterization at the level of composition and topography of the Oxtein^®^ dental implants was analyzed. In our SEM analysis, we observed a homogeneous surface with good mechanical finish and few, if, any impurities according to the result obtained with XPS.

The main components of the surface of the dental implant were Ti, O, and C. In addition, other elements, such as P, Ca, Si, N, Cl, etc., were also observed. Normally, they are presented in low percentages—a few units at most—and may be derived from washing or, as the case may be, from some of the processing phases [26]. The surface elements of the implants of the present study were mainly Ti, O, and C. These data are consistent with previous studies [25,27].

Vazouras et al. performed a study of SLA surface composition of Straumann^®^ (Basel, Switzerland) implants whose components were (expressed in atomic percentage): O 64.12%; Ti 13.73%; C 20.87%; and N 0.86%. 25 Kang et al. also analyzed this surface, in which they obtained as a result: O: 47.1%; Ti: 20.1%; C: 32%; and N: 1% [20].

An implant only has a surface free of impurities when it is subjected to vacuum because once the surface of the implant comes into contact with the air, an oxide layer is formed that covers it. This layer, which is nontoxic, is based on TiO, TiO_2_, and TiO_3_. The maximum percentage of Ti that can theoretically be observed through its analysis is approximately 33% [28]. Usual value of Ti is between 14% and 19%. A percentage of Ti greater than 12% can be considered satisfactory, as was the case with our samples, in which a value of 18.0 ± 0.1% was obtained. It is, therefore, an expected value, which is in accordance with the literature and which will provide good resistance to corrosion and promote osseointegration [25,29,30,31].

The type of titanium used is another important factor that must be added to the variables of surface characterization, as it directly influences the roughness of the implant due to different hardness of titanium according to its class [32]. In our implants, the titanium used was grade IV.

According to the literature, the maximum percentage of bone-implant contact is approximately 60%, and this is due to an incomplete gear between both that can be favored, among other factors, by the inevitable deposition of carbon atoms on the surface of the implant, which affects its bioactivity and is the biggest pollutant [33,34].

Ti constantly absorbs organic impurities from the atmosphere, water, or cleaning solutions. The percentage of carbon found according to the literature oscillates between 17.9% and 76.5% regardless of the topography of the surface [35]. The expected percentages of carbon in a titanium surface are between 30% and 40% [25,29]. The value obtained in our samples was 39.4 ± 0.5%, which means that osseointegration will not be affected by this percentage of carbon, and therefore, a high degree of bone-implant union is expected.

Hayashi et al. carried out a study on titanium discs in which contamination was caused naturally by carbon atoms at different concentrations. C/Ti (carbon on titanium) was successfully simulated. Subsequently, osteoblasts were grown on these surfaces. A decrease was observed at the level of osteoconductivity, cellular spread, protein adsorption, and cellular anchoring to the surface, as the concentration was higher C/Ti. This demonstrated that the carbon absorbed by the titanium surface is crucial for determining the initial affinity of the osteoblasts, and therefore, the importance of a low C/Ti ratio to increase osteoblastic activity on the titanium surface and thus obtain a higher integration [33]. Other authors, through their research, have obtained similar results [36,37,38,39].

The contamination of the surface by carbon particles, referred to in the literature as “biological aging of the surface”, is a very rapid and progressive phenomenon over time [40,41]. However, it has been observed that those implants that are in recent contact with the atmosphere have better properties in terms of cell and protein adhesion than those whose surface has been exposed for four weeks or more [30].

This contamination can be derived from different substances used during the manufacture of the implant or during the acid passivation process. In our case, the method used for the decontamination of the surface of the implant prior to its sterilization was the treatment with argon plasma [23].

Kang et al. conducted a comparative study of the chemical composition and morphology of different implant manufacturers comparing the surface area of the factory and the clean surface with argon plasma [20]. The global percentage of carbon detected initially was between 32% and 52%, which, after cleaning, decreased to 20% [20]. Larsson also carried out a similar study in which he obtained an atomic percentage of carbon lower than 10–15% [42].

Henningen et al. conducted a study of the surface treatment with argon plasma to observe if protein adsorption and the cellular anchor were related to the presence—or not—of carbon particles remaining on the surface. The osteoblastic cells cultured on the treated surfaces were larger and more elongated after the argon treatment. Therefore, it was concluded that this SLA treatment moderately improved rough surface conditions [43].

Argon plasma allows modification of the physiochemical and biological properties of treated surfaces, and therefore, their interaction with the environment. The cleaning of carbon impurities gives titanium implants a surface of greater hydrophilicity, and therefore, is more bioactive to favor the spreading, anchoring, and adhesion at the protein and cellular levels. All this can translate into an acceleration in osseointegration [39].

Further, in addition to decontaminating, argon plasma plays an antibacterial role. Annunziata et al. dipped discs with different surface treatments in human saliva. Two decontamination methods were used: One with phosphate-buffered saline (PBS) washing and subsequent centrifugation and one directly treating the surface with argon plasma. In the latter method, a cycle of 12 min was sufficient to eliminate any traces of bacterial presence, independent of the roughness of the surface (there were no significant differences). However, in the first method, bacterial colonies were detected after treatment [24].

Canullo et al. evaluated and verified the biological potential of the use of argon plasma in terms of osteoblastic, fibroblastic cell adhesion, and protein adsorption [42,44,45]. In another comparative analysis, argon plasma was superior with respect to not decontaminating the surface and also against other decontamination methods, such as steam [46].

The handling of the implant must be taken into account, as it influences primary stability and osseointegration, as analyzed by Da Costa et al. in their study [25]. They evaluated the possible morphological and compositional changes when inserting and removing the implants of a polyurethane bone model. No morphological changes were observed, only changes in the amount of organic material particles. In the implants with acid passivation treatment, there was a reduction in the amount of Ti with respect to the mechanized implants, which is important to bear in mind, as high amounts of Ti in the oxide layer favor osseointegration. In addition, implants with surface treatment accumulated a greater quantity of carbon particles, whose influence on osseointegration has been previously mentioned.

While implants with a rough surface induce osseointegration better than machined ones, it is important that they be manipulated carefully, as this surface treatment can have a negative influence on changes in the surface of titanium oxide [25].

Surface roughness is a factor that has a determining influence on the balance between bone formation and resorption on the bone-implant interface, and therefore, on its stability [12,13,14,25]. Through the surface treatment of an implant, its structure is modified, its roughness increases, and greater osseointegration is obtained. This conclusion has been reached by several authors who obtained better results in terms of surface characteristics compared to machined surfaces [15,17].

In our implants, the treatment was blasting with coarse grain (250–500 μm) and double acid passivation, a surface previously mentioned as SLA, which presents different topographies at different scales within the same surface, as can be observed in the SEM images included in this study [12].

The optimum value of average roughness found in the literature is 1–2 μm, which coincides with our average roughness value obtained of 1.51 μm ± 0.22. This value is similar to those of the SLA surface of Straumann^®^ implants collected in the literature, which are between 1.19 μm ± 0.04 and 1.53 μm ± 0.11. This short range of roughness due to etching shows a typical tip-to-tip distance below the size of the bone cell, which creates a three-dimensional sponge appearance that is very effective in promoting osseointegration, wetting by the blood, the formation of clots, and the release of the growth factor thanks to the activation of the platelets [47,48]. On the surfaces analyzed in our study with SEM, this three-dimensional “spongy” structure was observed.

Although in the literature there is a good correlation between average R_a_ rugosity and greater implant anchoring, it cannot be extrapolated to biological behavior until the capacity for osseointegration is demonstrated. Clinical behavior cannot be extrapolated based solely on the roughness descriptive parameters. Several authors and works have shown that the R_a_ value is insufficient by itself to characterize a given surface [32].

In relation to our study, the biological data of the rough surface tested indicate that there was an improvement in osteocalcin expression at 48 h and 96 h. Regarding the expression of collagen type I, there were no significant differences between both surfaces. In relation to the expression of alkaline phosphatase, these were better in the microrough surface only in the 96 h of culture. Cell proliferation was in almost all similar data on both surfaces. This viability data provide show that ALP and Collagen I expression is not correlated with the viability of cells.

Lukaszewska-Kuska et al. conducted a comparative study of several modified Ti surfaces (including SLA) analyzing the effects of surface roughness and chemical composition on the vitality, morphology, and orientation of human osteoblasts. They observed that surface roughness patterns influence orientation in growth. The rough samples were isotropic and were not oriented with respect to the surface characteristics (unlike the smooth surfaces, in which the cells grow along the grooves created after machining) [49].

They also observed that surface topography influences osteoblastic morphology; on rough surfaces, cells have a smaller coverage area and are less dispersed than on smooth surfaces. However, they exhibit numerous cytoplasmic extensions, phylodapy, and interconnections, which indicate greater adhesion properties compared to smooth surfaces. An increase in cellular vitality was observed over time on rough surfaces with respect to smooth surfaces [49].

Lagonegro et al. compared the distribution of osteoblastic cells in machined titanium discs and SLA discs. The arrangements of the cells on this last surface were elongated projections of the cytoplasm that followed the surface of the peaks and avoided the valleys of the surface. They showed that when cells grow on microtextured substrates, such as SLA, its adhesion area is limited to the Ti peaks of the surface and the surface areas near the peaks, and the cell bodies join over the valleys of the surface [50].

Blatt et al. concluded in their study of early cellular response that the different surfaces of implants influence expression patterns in terms of proliferation, adherence, and differentiation in early osteoblastic maturation [51,52]. With regard to the surface obtained from the combination of blasting and acid passivation, several authors have suggested that when greater roughness is obtained, greater cell adhesion, and acceleration occurs in early osseointegration (first week) [53,54].

New frontiers and challenges of Ti implants have been addressed to improvement of bioactivity, fighting of bacterial infection and biofilm formation, as well as modulation of inflammation. This is closely related to the clinical demand of multifunctional implants able to simultaneously have a number of specific responses with respect to body fluids, cells (osteoblasts, fibroblasts, macrophages), and pathogenic agents (bacteria, viruses) [55]. These are the new lines that must be addressed in the field of implant biomaterials.

## 4. Materials and Methods

Four Oxtein^®^ dental implants (Zaragoza, Spain) were investigated with the following coding: Code L63713T (titanium grade IV, 3.75 mm diameter, 13 mm length). The surface of the implants was SLA type obtained from coarse-grained, double passivated acid and decontaminated with argon plasma. The samples were in their sealed packages and were opened in our laboratory. To characterize the chemical composition of the surface, the X-ray photoelectron spectroscopy (XPS) technique was used, and to perform the topographic surface evaluation, the scanning electronic microscope (SEM) technique was used, as described in the following sections.

### 4.1. Chemical Analysis of the Surface

Surface composition analysis was performed through XPS (PHI 5400 ESCA Perkin Elmer, USA) equipped with an X-ray source with an MG anode maintained at 20 kV with a nominal power rating of 200 W. The depth that can be reached is approximately 5 mm. The pressure inside the analysis chamber was maintained at approximately 10^−9^ torr. The XPS spectrum was obtained at C 1s, Ti 2p, N 1s, and C 1s. The results of the analysis are expressed in atomic percentages. The XPS analysis was carried out on two samples that were carefully extracted from their packaging and immediately deposited in the container for the sample of the instrument. To prevent accidental contamination, all procedures were conducted with the greatest possible care.

### 4.2. Surface Topography

The surface topography of the implants tested was evaluated by means of an SEM (EVO MA 10 SEM, Carl Zeiss, Germany). The acceleration voltage of the electron was maintained at 20 kV, and the working distance was between 11 and 13.5 mm. These parameters are shown in the images together with the degree of magnification (MAG).

The roughness was assessed quantitatively by stereo SEM (SSEM) in accordance with ISO 4287 using software designed to convert conventional SEM images into three-dimensional data (Mex 6.0, Alicona Images, Chicago, IL, USA). All parameters defined in the standard have been given value. In particular, this evaluation takes advantage of the basic principle of stereoscopic vision. In essence, two images of the same field of vision are acquired after the 2000× eccentric rotation through a five-degree inclination angle. This is obtained by changing the angle between the sample and the electrons by tilting the table that holds the sample. The degree of inclination is established and controlled by the instrument control software.

The pair of images obtained (stereopar), the size of the field of view, and the angle of inclination are the useful data that the software converts into a simple three-dimensional image, where each data point is characterized by the values of the X, Y, and Z coordinates. The image obtained through this process allows researchers to measure the height profiles (roughness profiles) and calculate the different roughness parameters defined by the relevant bibliography and standards. Additional details about this process are provided in the results section.

The quantitative roughness parameters used in this study are: R_a_ (mean profile roughness); R_q_ (roughness of the mean square value of the profile); R_z_ (maximum height of the profile roughness); R_p_ (maximum height of the roughness peak of the profile); R_v_ (maximum depth of the valley of the profile roughness); and R_c_ (average height of irregularities of the roughness profile).

### 4.3. Cell Culture

Tests were performed on grade 4 Ti disks (6 mm diameter) subjected to the same surface treatment as OXTEIN implants while machined disks were used as a control. Tests were performed using the widely investigated continuous cell line SaOS-2 (BS-TCL-90) human osteosarcoma osteoblast-like cells, purchased from “Centro Substrati Cellulari dell’Istituto Zooprofilattico Sperimentale della Lombardia e dell’Emilia Romagna”.

The following culture protocol was used: A suspension of 1.05 ± 0.13 × 105 SaOS-2 cells (obtained by adding 2 mL of trypsin/EDTA solution to the monolayer inside a T75 Falcon flask) in 2.5 mL of McCoy’s 5A medium, supplemented with 15% foetal calf serum, L-glutamine, penicillin, streptomycin and amphotericin B (all purchased from GIBCO, INVITROGEN Srl, San Giuliano Milanese) was introduced into sterile 12-well polystyrene culture plates (12-well multiwell plates, Cell Star, Greiner One™), one for every experimental time, containing test and control samples. For each experimental time four replicates (that is, four treated and four machined disks) were used, within each microplate the four remaining microwells were seeded with cells and used as a visual control of cell growth. The culture plates were then placed in an incubator at 37 °C, with 5% CO_2_ and 98% relative humidity. Samples were removed from the multiwell at a given experimental time (24, 48, and 72 h), delicately washed with Dulbecco’s Phosphate Buffered Saline (DPBS, Gibco, INVITROGEN Srl), in order to remove any non-adhered cells and analyzed through RT-PCR, as described in the next section

### 4.4. RT-PCR Measurement

The expression of cell differentiation markers was assessed using the real time reverse transcription polymerase chain reaction (qRT-PCR). SaOS2 cells were cultured on the test samples and total RNA was extracted at given time points using MagMax Total RNA Isolation Kit (Applied Biosystems) following the manufacturer’s instructions. RNA quality was assessed by checking the A260/A280 ratio (1.6–2.0).

Then total RNA was used as a template for cDNA synthesis using random hexamers as primer and Multiscribe Reverse Transcriptase (High Capacity cDNA RT Kit from Applied Biosystems). The following osteogenic markers were used: Collagen I (CollI); Alkaline Phosphatase (ALP); Osteocalcin (OCN). GADPH expression was used as a reference. cDNA amplification and relative gene quantification was performed using Taq Man probe and primers from Applied Biosystems (Hs 00266705_g1, GAPDH; Hs 00164004_m1, Coll I; Hs 01029144_m1, ALP; Hs 00609452_g1, OCN). Real time PCR was performed in quadruplicate for all samples and targets on a Step-One instrument (Applied Biosystems, Madrid, Spain) using the software Step-One, version 2.1. PCRs were carried out in a total volume of 20 μL and the amplification was performed as follows: After an initial denaturation at 95 °C for 10 min, the PCR was run for 40 cycles at 95 °C for 15 s and at 60 °C for 1 min.

To normalize the content of cDNA samples, the comparative threshold (*C*_t_) cycle method, consisting on the normalization of the number of target gene copies versus the endogenous reference gene GAPDH, was used. The Ct is defined as the fractional cycle number at which the fluorescence generated by the cleavage of the probe passes a fixed threshold baseline when amplification of the PCR product is first detected. For comparative analysis of gene expression, data were obtained using the Δ*C*_t_ method.

### 4.5 Evaluation of SaOS2 growth and viability through the MTT test.

A suspension of 1.05 ± 0.13 × 10^5^ SaOS2 cells (obtained by adding 2 mL of trypsin/EDTA solution to the monolayer inside a T75 Falcon flask) in 2.5 mL of McCoy’s 5A medium, supplemented with 15% foetal calf serum, L-glutamine, penicillin, streptomycin and amphotericin B (all purchased from Gibco, INVITROGEN Srl, San Giuliano Milanese) was introduced into sterile 6-well polystyrene culture plates (6-well multiwell plates, Cell Star, Greiner One™), together with the samples (three replicates for each experimental time for each surface). At selected time intervals (24, 48, and 96 h), cell viability was evaluated by the MTT test (Sigma Aldrich, Germany), through the measurement of cells succinate dehydrogenase (SDH) activity. SDH is a key enzyme of the Krebs cycle, because it catalyzes the conversion from succinic acid to fumaric acid; its evaluation by biochemical means is commonly used to check cell’s health. Briefly, cells were washed twice with sterile DPBS, and then the DPBS was replaced with 1 mL/well of MTT (3-[4,5-dimethylthiazol-2-yl]-2,5-diphenyl tetrazolium bromide)-sodium succinate solution (5 mg/mL solution). The cells and MTT solution were incubated at 37 °C for 3 h in the incubator. During this time, yellow water-soluble MTT solution is transformed by the cells mitochondrial dehydrogenase into a blu-violet insoluble formazan. By measuring the amount of blu-violet formazan produced it is possible to measure mitochondrial activity, and as a consequence, cell viability. At the end of the incubation period, the MTT solution was removed and replaced with 1 mL/well of isopropanol analytical grade (Sigma Aldrich, Schnelldorf, Germany). The wells were swirled until the purple color was even and the absorbance was evaluated at 570 nm using as Tecan microplate reader (Tecan, Mendendorf, Switzerland).

## 5. Conclusions

Considering the data obtained in our study of Oxtein^®^ implants, which have been analyzed and discussed based on numerous published studies, we indicate three primary points: (1) The total carbon content and the relative concentration of titanium are in accordance with those found in the literature and are suitable for the use of said surface in dental therapeutics; (2) Other detected elements conform to implant surface data provided by other authors in the literature and are within the expected concentration from a quantitative point of view, completely fulfilling their designed use; and (3) The roughness of the treatment performed on the implants throws a spongy, three-dimensional surface suitable for bone growth on it. (4) The biological properties of the microrough surface improve the cellular development and protein expression that can lead to a better osseointegration in this surface. All of these results point to an adequate surface treatment of Oxtein^®^ implants for clinical use in humans.

## Figures and Tables

**Table 1:** MTT (3-(4,5-dimethylthiazol-2-yl)-2,5-diphenyltetrazolium bromide; cell proliferation test) measurement as absorbance values at 570 nm; *p* > 0.05 for all values except for values at 24 h (*p* < 0.05).

Time	Machined Surface	Microrough Surface
24 h	0.105 ± 0.005	0.153 ± 0.016
48 h	0.285 ± 0.003	0.294 ± 0.010
96 h	0.318 ± 0.017	0.324 ± 0.017

## References

[1] Branemark P.I., Adell R., Breine U., Undstrom J., Hallen O., Ohman A. (1969). Intra-osseous anchorage of dental prostheses I. Experimental studies. Scand. J. Plast. Reconstr. Surg..

[2] Hanawa T. (1999). In vivo metallic biomaterials and surface modification. Mater. Sci. Eng. A.

[3] Esposito M., Ardebili Y., Worthington H.V. (2014). Interventions for replacing missing teeth: Different types of dental implants. Cochrane Database Syst. Rev..

[4] Najeeb S., Zafar M.S., Khurshid Z., Siddiqui F. (2016). Applications of polyetheretherketone (PEEK) in oral implantology and prosthodontics. J. Prosthodont. Res..

[5] Najeeb S., Khurshid Z., Zohaib S., Zafar M.S. (2016). Bioactivity and osseointegration of PEEK are inferior to those of titanium: A systematic review. J. Oral Implant..

[6] Elias C.N., Fernandes D.J., Resende C.R., Roestel J. (2015). Mechanical properties, surface morphology and stability of a modified commercially pure high strength titanium alloy for dental implants. Dent. Mater..

[7] Buser D., Nydegger T., Hirt H.P., Cochran D.L., Nolte L.P. (1998). Removal torque value of titanium implants in the maxilla of miniature pigs. Int. J. Oral Maxillofac. Implant..

[8] Wennerberg A., Albrektsson T., Lausmaa J. (1996). Torque and histomorphometric evaluation of c.p. titanium screws blasted with 25- and 75-um-sized particles of Al_2_O_3_. J. Biomed. Mater. Res..

[9] Brunette D.M., Tengvall P., Textor M., Thomsen P. (2011). Titanium in Medicine: Material Science, Surface Science, Engineering, Biological Responses and Medical Applications.

[10] Lausmma J., Brunette D.M., Tengvall P., Textor M., Thomsen P. (2011). Mechanical, Thermal, chemical and Electrochemical Surface Teatment of Titanium. Titanium in Medicine: Material Science, Surface Science, Engineering, Biological Responses and Medical Applications.

[11] Bagno A., di Bello C. (2004). Surface Teatment and Roughness Properties of Ti-based Biomaterials. J. Mater. Sci. Mater. Med..

[12] Bauer S., Schmuki P., von der Mark K., Park J. (2012). Engineering biocompatible implant surfaces. Part I: Materials and surfaces. Prog. Mater. Sci..

[13] Wennerberg A., Albrektsson T. (2010). On implant surfaces: A review of current knowledge and opinions. Int. J. Oral Maxillofac. Implant..

[14] Sezin M., Croharé L., Ibañez J.C. (2016). Microscopic Study of Surface Microtopographic Characteristics of Dental Implants. Open Dent. J..

[15] Abrahamsson I., Zitzmann N.U., Berglundh T., Wennerberg A., Lindhe J. (2001). Bone and soft tissue integration to titanium implants with different surface topography: An experimental study in the dog. Int. J. Oral Maxillofac. Implant..

[16] Buser D., Schenk R.K., Steinemann S., Fiorellini J.P., Fox C.H., Stich H. (1991). Influence of surface characteristics on bone integration of titanium implants. A histomorphometric study in miniature pigs. J. Biomed. Mater. Res..

[17] Lukaszewska-Kuska M., Leda B., Gajdus P., Hedzelek W. (2017). Evaluation of modified titanium surfaces physical and chemical characteristics. Nucl. Instrum. Methods Phys. Res. Sect. B Beam Interact. Mater. Atoms..

[18] Albrektsson T., Wennerberg A. (2004). Oral implant surfaces: Part 1—Review focusing on topographic and chemical properties of different surfaces and in vivo responses to them. Int. J. Prosthodont..

[19] Rosa M.B., Albrektsson T., Francischone C.E., Schwartz Filho H.O., Wennerberg A. (2012). The influence of surface treatment on the implant roughness pattern. J. Appl. Oral Sci..

[20] Kang B.S., Sul Y.T., Oh S.J., Lee H.J., Albrektsson T. (2009). XPS, AES and SEM analysis of recent dental implants. Acta Biomater..

[21] Mendoza-Arnau A., Vallecillo-Capilla M.F., Cabrerizo-Vílchez M.Á., Rosales-Leal J.I. (2016). Topographic characterisation of dental implants for commercial use. Med. Oral Patol. Oral Cir. Bucal.

[22] Wennerberg A., Albrektsson T. (2009). Effects of titanium surface topography on bone integration: A systematic review. Clin. Oral Implant. Res..

[23] Cools P., Geyter N.D., Vanderleyden E., Dubruel P., Morent R. (2014). Surface Analysis of Titanium Cleaning and Activation Processes: Non-thermal Plasma Versus Other. Plasma Chem. Plasma Process..

[24] Annunziata M., Canullo L., Donnarumma G., Caputo P., Nastri L., Guida L. (2016). Bacterial inactivation/sterilization by argon plasma treatment on contaminated titanium implant surfaces: In vitro study. Med. Oral Patol. Oral Cir. Bucal..

[25] Lima da Costa Valente M., Shimano A.C., Marcantonio Junior E., Reis A.C. (2015). Relationship between the surface chemical composition of implants and contact with the substrate. J. Oral Implant..

[26] Lausmaa J. (1996). Surface spectroscopic characterization of titanium material. J. Electr. Spectr. Rel. Phen..

[27] Bruschi M., Steinmüller-Nethl D., Goriwoda W., Rasse M. (2015). Composition and Modifications of Dental Implant Surfaces. J. Oral Implant..

[28] Vazouras K. (2013). Surface Analysis of SLA and SLActive Dental Implants. Ph.D. Thesis.

[29] Albrektsson T., Brånemark P.I., Hansson H.A., Lindström J. (1981). Osseointegrated titanium implants. Requirements for ensuring a long-lasting, direct bone-to-implant anchorage in man. Acta Orthop. Scand..

[30] Roy M., Pompella A., Kubacki J., Szade J., Roy R.A., Hedzelek W. (2016). Photofunctionalization of titanium: An alternative explanation of its chemical-physical mechanism. PLoS ONE.

[31] Li C., Monti S., Carravetta V. (2012). Journey toward the Surface: How Glycine Adsorbs on Titania in Water Solution. J. Phys. Chem. C.

[32] Chrcanovic B.R., Wennerberg A., Martins M.D. (2015). Influence of Temperature and Acid Etching Time on the Superficial Characteristics of Ti. Mater. Res..

[33] Hayashi R., Ueno T., Migita S., Tsutsumi Y., Doi H., Ogawa T., Hanawa T., Wakabayashi N. (2014). Hydrocarbon Deposition Attenuates Osteoblast Activity on Titanium. J. Dent. Res..

[34] Ogawa T., Nishimura I. (2003). Different bone integration profiles of turned and acid-etched implants associated with modulated expression of extracellular matrix genes. Int. J. Oral Maxillofac. Implant..

[35] Morra M., Cassinelli C., Bruzzone G., Carpi A., Di Santi G., Giardino R., Fini M. (2003). Surface chemistry effects of topographic modification of titanium dental implant surfaces: 1. Surface analysis. Int. J. Oral Maxillofac. Implant..

[36] Ishijima M., Soltanzadeh P., Hirota M., Tsukimura N., Shigami T., Ogawa T. (2015). Enhancing osteoblast-affinity of titanium scaffolds for bone engineering by use of ultraviolet light treatment. Biomed. Res..

[37] Ueno T., Ikeda T., Tsukimura N., Ishijima M., Minamikawa H., Sugita Y., Yamada M., Wakabayashi N., Ogawa T. (2016). Novel antioxidant capability of titanium induced by UV light treatment. Biomaterials.

[38] Ogawa T. (2014). Ultraviolet photofunctionalization of titanium implants. Int. J. Oral Maxillofac. Implant..

[39] Yoshihara C., Ueno T., Chen P., Tsutsumi Y., Hanawa T., Wakabayashi N. (2017). Inverse response of osteoblasts and fibroblasts to growth on carbon-deposited titanium surfaces. J. Biomed. Mater. Res. B Appl. Biomater..

[40] Roy M., Hędzelek W. (2014). Photofunctionalization: A new method to bio-activate the titanium implant surface—Review of literature. Czas. Stomatol..

[41] Choi S.H., Jeong W.S., Cha J.Y., Lee J.H., Lee K.J., Yu H.S., Choi E.H., Kim K.M., Hwang C.J. (2017). Overcoming the biological aging of titanium using a wet storage method after ultraviolet treatment. Sci. Rep..

[42] Larsson Wexell C., Thomsen P., Aronsson B.O., Tengvall P., Rodahl M., Lausmaa J., Kasemo B., Ericson L.E. (2013). Bone response to surface-modified titanium implants: Studies on the early tissue response to implants with different surface characteristics. Int. J. Biomater..

[43] Henningsen A., Smeets R., Hartjen P., Heinrich O., Heuberger R., Heiland M., Precht C., Cacaci C. (2017). Photofunctionalization and non-thermal plasma activation of titanium surfaces. Clin. Oral Investig..

[44] Canullo L., Genova T., Tallarico M., Gautier G., Mussano F., Botticelli D. (2016). Plasma of Argon Affects the Earliest Biological Response of Different Implant Surfaces. J. Dent. Res..

[45] Canullo L., Cassinelli C., Götz W., Tarnow D. (2013). Plasma of argon accelerates murine fibroblast adhesion in early stages of titanium disk colonization. Int. J. Oral Maxillofac. Implant..

[46] Canullo L., Peñarrocha-Oltra D., Marchionni S., Bagán L., Peñarrocha-Diago M.A., Micarelli C. (2014). Soft tissue cell adhesion to titanium abutments after different cleaning procedures: Preliminary results of a randomized clinical trial. Med. Oral Patol. Oral Cir. Bucal..

[47] Szmukler-Moncler S., Perrin D., Ahossi V., Magnin G., Bernard J.P. (2004). Biological properties of acid etched titanium implants: Effect of sandblasting on bone anchorage. J. Biomed. Mater. Res. B Appl. Biomater..

[48] Kim H., Choi S.H., Ryu J.J., Koh S.Y., Park J.H., Lee I.S. (2008). The biocompatibility of SLA-treated titanium implants. Biomed. Mater..

[49] Lukaszewska-Kuska M., Wirstlein P., Majchrowski R., Dorocka-Bobkowska B. (2018). Osteoblastic cell behaviour on modified titanium surfaces. Micron.

[50] Lagonegro P., Trevisi G., Nasi L., Parisi L., Manfredi E., Lumetti S., Rossi F., Macaluso G.M., Salviati G., Galli C. (2017). Osteoblasts preferentially adhere to peaks on micro-structured titanium. Dent. Mater. J..

[51] Blatt S., Pabst A.M., Schiegnitz E., Hosang M., Ziebart T., Walter C., Al-Nawas B., Klein M.O. (2017). Early cell response of osteogenic cells on differently modified implant surfaces: Sequences of cell proliferation, adherence and differentiation. J. Craniomaxillofac. Surg..

[52] Yokose S., Klokkevold P.R., Takei H.H., Kadokura H., Kikui T., Hibino Y., Shigeta H., Nakajima H., Kawazu H. (2017). Effects of surface microtopography of titanium disks on cell proliferation and differentiation of osteoblast-like cells isolated from rat calvariae. Dent. Mater. J..

[53] Wei N., Bin S., Jing Z., Wei S., Yingqiong Z. (2014). Influence of implant surface topography on bone-regenerative potential and mechanical retention in the human maxilla and mandible. Am. J. Dent..

[54] Herrero-Climent M., Lázaro P., Vicente Rios J., Lluch S., Marqués M., Guillem-Martí J., Gil F.J. (2013). Influence of acid-etching after grit-blasted on osseointegration of titanium dental implants: In vitro and in vivo studies. J. Mater. Sci. Mater. Med..

[55] Spriano S., Yamaguchi S., Baino F., Ferraris S. (2018). A critical review of multifunctional titanium surfaces: New frontiers for improving osseointegration and host response, avoiding bacteria contamination. Acta Biomater..

